# PPARs and Mitochondrial Metabolism: From NAFLD to HCC

**DOI:** 10.1155/2016/7403230

**Published:** 2016-12-27

**Authors:** Tommaso Mello, Maria Materozzi, Andrea Galli

**Affiliations:** ^1^Clinical Gastroenterology Unit, Department of Biomedical Clinical and Experimental Sciences “Mario Serio”, University of Florence, Viale Pieraccini 6, 50129 Florence, Italy; ^2^Careggi University Hospital, Florence, Italy

## Abstract

Metabolic related diseases, such as type 2 diabetes, metabolic syndrome, and nonalcoholic fatty liver disease (NAFLD), are widespread threats which bring about a significant burden of deaths worldwide, mainly due to cardiovascular events and cancer. The pathogenesis of these diseases is extremely complex, multifactorial, and only partially understood. As the main metabolic organ, the liver is central to maintain whole body energetic homeostasis. At the cellular level, mitochondria are the metabolic hub connecting and integrating all the main biochemical, hormonal, and inflammatory signaling pathways to fulfill the energetic and biosynthetic demand of the cell. In the liver, mitochondria metabolism needs to cope with the energetic regulation of the whole body. The nuclear receptors PPARs orchestrate lipid and glucose metabolism and are involved in a variety of diseases, from metabolic disorders to cancer. In this review, focus is placed on the roles of PPARs in the regulation of liver mitochondrial metabolism in physiology and pathology, from NAFLD to HCC.

## 1. Introduction

Liver cancer is a major challenge in contemporary medicine. It represents the fifth most common cancer in men, the ninth in women, and the second most frequent cause of mortality among oncological patients. It was responsible for nearly 746,000 deaths in 2012, with an estimated incidence of over 780,000 new cases yearly worldwide [1]. The prognosis for liver cancer is extremely poor (overall ratio of mortality to incidence of 0.95), reflecting the absence of effective treatments. The most frequent type of primary liver cancer is hepatocellular carcinoma (HCC), accounting for up to 85% of total cancers [2].

Major risk factors include HBV or HCV infection, alcoholic liver disease, and most likely nonalcoholic liver disease (NAFLD) [2]. These and other chronic liver diseases lead to cirrhosis, which is found in 80–90% of HCC patients [2]. NAFLD is now the most common liver disease worldwide [3], with a global prevalence of about 25%. NAFLD is closely associated with other metabolic disorders such as obesity, metabolic syndrome, and type 2 diabetes [3]. Indeed, obesity and diabetes are now definitively recognized as independent risk factors for the development of HCC [4, 5]. NAFLD is histologically classified into nonalcoholic fatty liver (NALF), defined as the presence of steatosis in the absence of causes for secondary hepatic fat accumulation (i.e., alcohol consumption, steatogenic drugs, or genetic disorders), and nonalcoholic steatohepatitis (NASH), in which steatosis is complicated by inflammation and hepatocellular damage (ballooning hepatocytes), with or without fibrosis [6]. A relatively small portion of NAFL patients evolve into NASH, a progressive type of liver disease with the potential of evolving into cirrhosis and HCC. The cumulative incidence of HCC in NASH cirrhosis ranges from 2.4% to 12.8%, and although it is lower than in HCV cirrhotic patients, the absolute burden of NASH related HCC is higher due to the epidemic spread of NAFLD [7]. Moreover, NAFLD greatly increases the risk of HCC from other aetiologies, especially HCV and HBV. While the vast majority of HCC arise in cirrhotic livers, it can also occur in noncirrhotic patients [2]. Of notice, a significant amount of new HCC cases is diagnosed in patients with noncirrhotic NASH [4, 8]. The global incidence of HCC among NAFLD patients was recently estimated to be 0.44 per 1,000 person-year [3], which combined with the epidemic spread of metabolic disorders results in an enormous burden. The recent meta-analysis by Younossi et al. raised the question whether NAFLD could even precede the onset of metabolic syndrome rather than just being the hepatic manifestation of it [3].

The mechanisms that promote HCC development in NASH/NAFLD patients are complex and still poorly understood. A number of molecular mechanisms have been linked to obesity and related metabolic disorders that may accelerate the development of HCC, such as adipose-derived inflammation, lipotoxicity, and insulin resistance. These and other pathological events in obesity have complex interactions while their relative contribution to hepatocarcinogenesis in various stages of NAFLD progression remains to be determined.

Mitochondria can be seen as the energetic hub of the cell. As such, beyond their role in energy production, they play a central role in coordinating the cell anabolic and catabolic processes, in balancing the cell energetic demands in response to internal and external stimuli, and in the regulation of several cell signaling pathways. Deregulation of mitochondrial activity is a common trait to several human diseases, including cancer. Since Warburg, it has long been known that cancer cells undergo a radical metabolic shift toward glycolysis, irrespective of the oxygen availability (aerobic glycolysis) [9]. However, the actual significance of this metabolic remodeling, its consequences on cancer cell biology, and its plasticity have begun to be grasped only in recent years. The initial perception of the Warburg effects was that cancer cells rely primarily on glycolysis for energy production due to a defective mitochondrial respiration [10]. On the contrary, it is now clear that cancer cells hijack their mitochondria metabolism toward massive anabolic processes, in order to cope with the cell fast-growing rates [11]. In this line of view, exacerbate biosynthesis, in particular lipid biosynthesis, rather than glycolysis dependence, emerges as cancer metabolic hallmark.

Peroxisome proliferator activated receptors (PPARs) are master regulators of whole body and liver metabolism. Despite a similar structure, the three PPAR isotypes *α*, *β*/*δ*, and *γ* vary greatly in tissue distribution, pharmacological and endogenous ligands, and biological effects. In the past decades PPARs have been the focus of massive research effort that helped uncovering their contribution to cancer, metabolic, and cardiovascular diseases. The different PPAR isotypes regulate lipid metabolism by a number of mechanisms: (i) controlling the rate of FA disposal through mitochondrial and peroxysomal *β*-oxidation (FAO), (ii) regulating lipid biosynthesis via de novo lipogenesis, (iii) regulating FA uptake in peripheral tissue and in the liver, (iv) regulating whole body lipid trafficking through apolipoproteins, (v) interacting in complex regulatory network with other nuclear receptors (LXR, FXR), coactivators (PGC-1*α* and *β*, SREBP), or corepressors (NCOR) involved in the metabolic homeostasis. As liver is primarily a metabolic organ, PPARs-regulated processes are involved virtually in any liver disease.

This review summarizes current notions on the roles of PPARs in the regulation of liver mitochondrial metabolism in physiology and pathology, from NAFLD to HCC.

## 2. PPARs and Mitochondrial Metabolism in the Liver

### 2.1. PPAR*α*

Peroxisome proliferator activated receptor *α* (PPAR*α*) is the main PPAR isotype expressed in the liver and plays a major role in energy homeostasis, by regulating lipid metabolism and ketone body formation [12]. In mice but not in humans, PPAR*α* also controls the glycolysis-gluconeogenesis pathway [13]. PPAR*α* natural ligands are endogenous lipids such as fatty acids (FA) and their derivatives (eicosanoids, oxidized phospholipids) [14], while synthetic ligands include the class of hypolipidemic drugs fibrates, xenobiotic agents, and plasticizers.

Despite the fact that FA and derivatives can bind and activate PPAR*α* in the liver, not all FA are created equal. Indeed, it has been now recognized that FA released in the bloodstream by the adipose tissue (i.e., during fasting or intense physical exercise) have little role as PPAR*α* agonist [15], while preferentially activating PPAR*β*/*δ*, whereas fatty acids derived from dietary intake or de novo lipogenesis are efficient PPAR*α* activators [15–18]. However, PPAR*α* is absolutely required for the metabolic adaptation to fasting, since PPAR*α* ^−/−^ mice, either full body [19] or liver-specific [20], develop steatosis with prolonged fasting. Moreover, the time course activation of PPAR*α* in the liver mimics the kinetics of circulating FFA during fasting, and liver transcriptomic profiling revealed that the fasted state (versus fed or refed) triggered the broader PPAR*α*-dependent response, strengthening the functional link between hepatic PPAR*α* and adipose tissue-FA disposal [20]. Since activation of hepatic PPAR*α* requires de novo lipogenesis [15, 21], the mechanisms that fine-tune PPAR*α* activation in different metabolic conditions are still unclear and possibly involve separate pools of PPAR*α* that can be activated in a context-dependent manner.

Moreover, the adipose tissue cross-talk with the hepatic PPARs can occur via adiponectin-induced FAO, which is dependent upon AdipoR2 subtype and requires PPAR*α* induction [22], and via FGF21, produced mainly in the liver in a PPAR*α*-dependent manner [20], which promotes both glucose uptake and lipolysis in the adipocytes [23], as well as hepatic lipid disposal [24].

**Figure 1:**
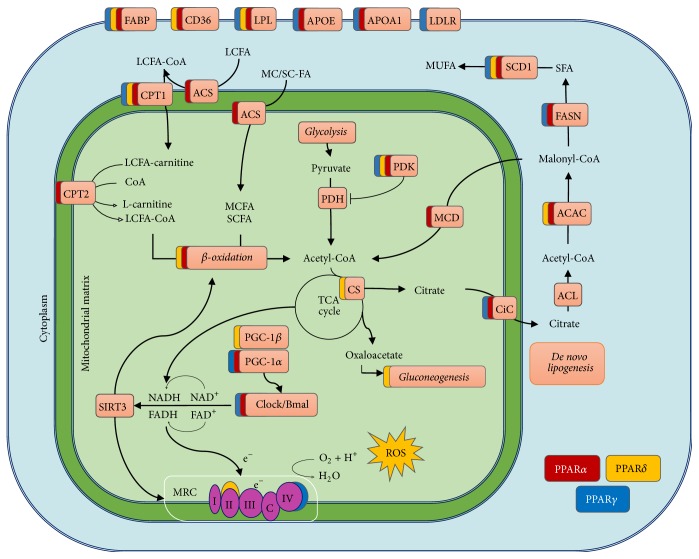
Role of hepatic PPARs in mitochondrial metabolism: fatty acid oxidation, circadian control of NAD+ dependent SIRT activity, de novo lipogenesis, and gluconeogenesis. Color-coding depicts PPAR isotypes-dependency of target genes.

In hepatocytes, PPAR*α* promotes the expression of several genes involved in FA uptake, activation to acyl-CoA, and transport to the mitochondria or peroxisomes and subsequent *β*- or *ω*-oxidation, ketogenesis, and lipoprotein trafficking [25, 26] (Figure 1).

Many of the PPAR*α* regulated genes directly modulate mitochondrial metabolism. Interestingly, among the many PPAR*α*-regulated genes in hepatocytes, those involved in mitochondrial metabolic functions, especially in fatty acid oxidation, are consistently dependent upon PPAR*α* regardless of the nutritional condition [20]. PPAR*α* target genes are also carnitine palmitoyl transferase 1 (CPT-1) and carnitine palmitoyltransferase 2 (CPT-2) [19, 25], which mediate transport of long-chain fatty acids through the outer and inner mitochondrial membrane, respectively, to initiate their degradation in the *β*-oxidative pathway (Figure 1). The *β*-oxidation cycle consists of four reactions, catalyzed by acyl-CoA dehydrogenases (ACADs), enoyl-CoA hydratases, L-3-hydroxyacyl-CoA dehydrogenase, and 3-ketoacyl-CoA thiolase, that sequentially remove two carbons—one acetyl-CoA molecule, until the acyl-CoA is completely converted to acetyl-CoA. The initial step of the *β*-oxidation cycle is catalyzed by length specific acyl-CoA dehydrogenases (such as ACADM, ACADS, and ACADVL), all of which are PPAR*α* target genes [26]. The last three steps are carried on by the mitochondrial trifunctional protein (MTP), a large complex of four *α* and four *β* subunits. The expression of both subunits (encoded by genes HADHA, HADHB) as well as the MTP protein 3-ketoacyl-CoA thiolase (encoded by ACAA2) is regulated by PPAR*α* [26].

The acetyl-CoA produced during FAO is then used to produce ketone bodies (acetoacetate and *β*-hydroxybutyrate) via mitochondrial HMG-CoA synthase, another PPAR*α* regulated gene [27]. Ketone bodies are released in the blood stream and, after conversion to citrate, fuel the TCA cycle in peripheral tissues (mostly heart, muscle, and brain).

FAO is functionally and physically linked to OXPHOS: the reducing equivalents produced by FAO are directly used by the electron transport chain (ETC); moreover, the two pathways are likely happening in large mitochondrial supercomplexes containing both FAO and ETC complexes [28]. Therefore, an unbalance in FAO or ETC directly affects the other pathway.

PPAR*α*, as well as *β*/*δ* and *γ*, also induces the expression of all uncoupling protein (UCP-1, UCP-2, and UCP-3), of which UCP-2 is the main type expressed in liver [29, 30]. Uncoupling proteins allow protons to reenter the mitochondrial matrix without production of adenosine triphosphate, thus promoting energy expenditure and FA oxidation.

Paralleling its role in promoting energy expenditure through FA disposal, PPAR*α* also inhibits the lipogenic pathway by induction of the malonyl-CoA decarboxylase which degrades malonyl-CoA, a precursor of FA biosynthesis and inhibitor of the mitochondrial transporter CPT-1 [31] (Figure 1).

### 2.2. PPAR*β*/*δ*

PPAR*β*/*δ* is ubiquitously expressed, often at higher level than PPAR*α* or *γ*. Overall, PPAR*β*/*δ* role in lipid metabolism appears to be largely overlapping with PPAR*α* in most tissues. Indeed, PPAR*β*/*δ* stimulates FAO in muscle and heart, the latter organ being extremely dependent on PPAR*β*/*δ* function [32].

Several PPAR*α* target genes are thus not surprisingly induced also by PPPAR*β*/*δ* (UCP-1, UCP-2, and UCP-3, FABP, FAT/CD36, LPL, ACS, and CPT-1) [33, 34] and loss of PPAR*α* in muscle is efficiently compensated by PPAR*β*/*δ* [33]. Indeed, numerous studies have shown that PPAR*β*/*δ* overexpression or activation in muscle dramatically improves FA utilization as energy source, reduces hyperlipidemia, improves endurance, and decreases insulin secretion from *β*-cells [32, 35–37].

However, in the liver PPAR*β*/*δ* seems to play a different role than PPAR*α*. Adenoviral-mediated overexpression of PPAR*β*/*δ* in the liver enhanced glucose utilization either to increase glycogen storage or to promote de novo lipogenesis, rather than inducing FAO [38] (Figure 1). PPAR*β*/*δ* induced the expression of several genes involved in glucose metabolism (GLUT2, GK, and pyruvate kinase) and lipogenesis (FAS, ACC1, ACC2, SCD1, SREBP-1c, and PGC-1*β*) [38]. Conversely, gluconeogenesis genes (PEPCK, HNF-4) were inhibited by PPAR*β*/*δ* expression in hepatocytes. Importantly, the levels of PPAR*α* and its target (CPT-1, acyl-CoA oxidase, and MCAD) were unaffected; therefore PPAR*β*/*δ* seems not to overlap with PPAR*α* function in the liver [38]. Consistently, whole transcriptome profiling and liver metabolites analysis of PPAR*α* ^−/−^ and PPAR*β*/*δ* ^−/−^ mice revealed clearly divergent roles [39]. Very interestingly, liver PPAR*β*/*δ* signals to PPAR*α* and activates FAO in muscle via the lipid molecule PC (18:0/18:1), whose production in the liver is PPAR*β*/*δ*-dependent [40].

Different roles for PPAR*α* and PPAR*β*/*δ* in mitochondriogenesis are also beginning to emerge. A transitory upregulation of PPAR*α*, and consequent induction of PGC-1*α*, is necessary to promote mitochondriogenesis in the early steps of differentiating embryonic stem cells. A robust upregulation of PPAR*β*/*δ* is instead needed to promote mitochondriogenesis at later stages of cells differentiation and correlates with the expression of mature hepatocytic markers [41].

Functional peroxisome proliferator response elements have been identified in the distal promoter of PGC-1*α*, providing the mechanistic basis for PPAR-induced mitochondrial biogenesis. However, the contribution of the diverse PPAR isotypes to PGC-1*α* induction appears to be cell context-dependent. PGC-1*α* is activated by PPAR*α* in brown adipose tissue [42] and by PPAR*γ* in both white and brown adipose tissue [42]. In skeletal muscle, PPAR*β*/*δ* but not PPAR*α* induce PGC-1*α* expression [43, 44].

In liver, PCG-1*α* is induced by fasting, paralleling PPAR*α* activation, and promotes gluconeogenesis, a process mediated by PPAR*β*/*δ* [45].

### 2.3. PPAR*γ*

PPAR*γ* is the main PPAR isotype expressed in white and brown adipose tissue. It is the master inducer of adipogenesis and promotes glucose uptake and utilization in the novo lipogenic pathway, therefore regulating whole body lipid metabolism and insulin sensitivity. Natural PPAR*γ* ligands are lipid molecules derivates, such as unsaturated FA, PGJ_2_, and oxidized LDL [14, 46, 47] while potent synthetic ligands include the insulin sensitizer class of drug TZD [48].

PPAR*γ* induces the expression of genes regulating glucose sensitivity (GLUT-4, IRS-1, IRS-2, and PI3K), as well as genes involved in FA uptake and mobilization (FAT/CD36, fatty acids binding proteins aP2, and lipoprotein lipase) and triglyceride synthesis (acyl-CoA synthetase, glycerol kinase, and PEPCK) [46, 49] (Figure 1). In the liver, PPAR*γ* is expressed in macrophages, endothelial cells, quiescent (nonactivated) stellate cells, and hepatocytes. Its complex actions on liver physiology are mostly mediated by its anti-inflammatory functions on macrophages and endothelial cells, antifibrotic function in hepatic stellate cells, and metabolic cross-talk between hepatocytes and adipocytes via FGF family members (FGF21, FGF-1). Mice with selective deletion of PPAR*γ* in hepatocytes developed relative fat intolerance, increased adiposity, hyperlipidemia, and insulin resistance. Loss of hepatic PPAR*γ* increased TG blood level and redistribution to other tissues, aggravating insulin resistance in muscle and adipose tissue [50, 51]. These models highlighted the role of liver PPAR*γ* in maintaining lipid/glucose homeostasis and insulin sensitivity.

PPAR*γ* also induces the expression of mitochondrial proteins, common to the other PPARs, such as CPT-1 and UCPs, suggesting a possible degree of overlap in mitochondrial metabolism regulation with other PPAR members. Probably the most relevant function of PPAR*γ* in mitochondria biology comes with its interaction with PGC-1 family members. PGC-1*α* was initially identified as a nuclear PPAR*γ* coactivator in mitochondrial rich brown adipose tissue-tissue [52]. Since then, it has become clear that PGC-1*α* and *β* control virtually any aspect of mitochondria function and biogenesis [53], by thoroughly coordinating a plethora of nuclear receptors (including all three PPARs, EER*α*) and nonnuclear receptor protein [54]. Indeed, PPAR*γ* can promote the expression of PGC-1*α*, which in turn potentiates PPAR*γ* activity [55]. Recently, steatogenic FA were shown to induce PPAR*γ* via PGC-1*α*, suggesting a link between mitochondria biogenesis and triglyceride accumulation [56].

## 3. Mechanisms of Mitochondrial Oxidative Stress Damage

Reactive oxygen species (ROS) are small reactive molecules generated by the normal cell metabolism, involved in homeostasis and signaling. ROS such as superoxide anion (O_2_^−^), hydrogen peroxide (H_2_O_2_), and hydroxyl radical (HO^•^) consist of radical and nonradical oxygen species formed by the partial reduction of oxygen. Cellular ROS levels are controlled by antioxidant systems such as reduced/oxidized glutathione (GSH/GSSG), reduced/oxidized cysteine (Cys/CySS), tioredoxin (Trx), peroxiredoxin (Prx), superoxide dismutase (SOD), and catalase.

An imbalance of the generation/neutralization of ROS, driven by an overproduction of ROS or a depletion of the antioxidant defenses, leads to a prooxidant state defined as “oxidative stress.” Oxidative stress can directly damage proteins, lipids, and DNA, leading to damaged macromolecules and organelles, but also deranges the redox circuits that regulate many signaling pathways [57]. In fact, while excessively high levels of oxidative stress lead the cell to apoptosis, a controlled increase of ROS serves as critical signaling molecules in cell proliferation and survival [58]. ROS can be generated by growth factor signaling through activation of the NADPH oxidase NOX1 or through the mitochondria. In turn, they can induce cellular signaling cascades by oxidation of phosphatases such as PTEN or PTP or kinases such as Src. This leads to the activation of several pathways such as a Src/PKD1-dependent NF-*κ*B activation mechanism, MAPK (Erk1/2, p38, and JNK), and the PI3K/Akt signaling. Aberrant levels of ROS induce a deregulation of these pathways, which are involved in several pathological conditions, such as NAFLD [59], diabetes [60], and cancer [58, 61].

Several different sources of ROS exist in mitochondria. ETC complex I and complex II, as well as other mitochondrial enzymes such as *α*-ketoglutarate dehydrogenase, pyruvate phosphate dehydrogenase, fatty acyl-CoA dehydrogenase, and glycerol 3-phosphate dehydrogenase, can produce O_2_^•−^ as byproduct, releasing it within the mitochondrial matrix. Moreover, H_2_O_2_ is produced by the monoamine oxidases (MAOs) located in the outer mitochondrial membrane [62, 63]. Therefore, mitochondria can produce a significant amount of ROS during OXPHOS and FAO, especially in the context of reduced antioxidant defense such as depletion of the mitochondrial glutathione pool [64].

Four main alterations are the direct result of ROS formation: lipid, protein and DNA oxidation, and depletion of antioxidant molecules.

Mitochondrial DNA (mtDNA) is particularly susceptible to oxidative damage due to the absence of protective histones, incomplete DNA repair mechanisms, and the close proximity to ROS production site, which increase the risk of double-strand breaks and somatic mutations with increased ROS production [65]. Since the ETC proteins are encoded exclusively in mtDNA, oxidative damage leads to defective mitochondrial respiration and to a second burst of ROS production that damages mitochondrial membrane and eventually results in loss of mitochondrial membrane potential and activation of proapoptotic pathways due to the ROS induced-ROS-release avalanche [64, 65]. Indeed, depletion and mutation of mtDNA have been described in several type of liver injury, including NASH [66].

Lipid peroxidation is the process under which lipids, mainly polyunsaturated fatty acids, are attacked by oxidants such as ROS. These reactions can form a variety of primary and secondary products, among which malondialdehyde (MDA) appears to be the most mutagenic and 4-hydroxynonenal (4-HNE) the most toxic. MDA induced mutations are involved in cancer and other genetic diseases. 4-HNE can also act as a signaling molecule modulating many pathways and inducing the expression of proteins, such as NF-*κ*B, Akt, MAPK, JNK, and PPARs. Lipid peroxidation occurs through a radical reaction; it is therefore extremely harmful to biological membranes where the damage can rapidly spread.

The depletion of mitochondrial ROS scavenger is a key step in the pathogenesis of ROS-related liver disease.

In NASH animal model, depletion of mGSH occurs due to cholesterol accumulation in the mitochondrial membrane [67] that disrupts the functionality of GSH transport from cytosol to mitochondria. Depletion of mGSH and other antioxidant systems are documented in NASH patients [68].

ROS can also act as second messengers in cellular signaling oxidizing proteins on cysteine residues, resulting in protein activation or inhibition. High levels of ROS can therefore activate pathways in a signal-independent manner and self-sustain many proproliferative pathways highly involved in cancer and liver diseases such as NASH/NAFLD.

For example, it has been demonstrated that ROS can directly oxidize and activate complexes such as inflammasomes: protein platforms that assemble in the presence of exogenous or endogenous danger signals such as pathogen associated molecular patterns (PAMPs) and damage-associated molecular patterns (DAMPs) to activate and amplify inflammatory pathways [69]. Typically, inflammasomes consist of a sensor (NLRs, ALRs, and TLRs), an adaptor (ASC), and the effector molecule caspase-1 [70]. Once caspase-1 is recruited and activated through autocatalytic cleavage by the inflammasome, it can proteolytically process the inflammatory cytokines IL-1*β* and IL-18 that lead to a specialized form of cell death called pyroptosis. Pyroptosis causes the release of IL-1*β* and amplify the inflammatory response downstream of inflammasome activation [70]. In the liver, inflammasomes are expressed in hepatocytes as well as in immune cells and can also be activated by fatty acids through a mechanism involving mitochondrial ROS, decreased autophagy, and IL-1*β* secretion. Inflammasomes are found overexpressed in NAFLD and NASH and their silencing reduced hepatic injury, steatosis, and fibrosis [69]. Interestingly, agonists of PPAR*β*/*δ* were shown to reduce fatty acid induced inflammation and steatosis by inhibiting inflammasomes [69, 71].

Lipid overload in NAFLD and NASH leads to mitochondrial dysfunction and increased oxidative stress, which results from both increased electron flux through the ETC and depletion of the mitochondrial antioxidant defense systems [64].

Reduced levels of GSH, SOD, and catalase as well as increased protein oxidation, a hallmark of increased oxidative stress, are found in NASH patients [68]. Consistently, the mitochondria of NASH patients have altered morphology [72, 73], reduced or mutated mDNA content [66], and reduced oxidative phosphorylation capacity [74]. Oxidative stress constitutes one of the key factors driving NAFLD progression to NASH [75]. Indeed, histological markers of oxidative stress, such as oxidized phosphatidylcholine, localize into steatotic/apoptotic hepatocytes and macrophages and correlate with the degree of steatosis [76].

Depletion of mtGSH and mitochondria oxidative damage are recapitulated also in several animal models of NASH. Interestingly, Llacuna and colleagues highlighted that mitochondrial damage in diverse animal models of NASH seemed to be dependent more on mitochondria cholesterol accumulation (ob/ob mice or HFD administration), rather than only fatty acid/triglyceride overload (choline deficiency model) [67]. Consistently, statins reduced mitochondrial damage in ob/ob mice and HFD models.

This line of view is confirmed by a recent report highlighting the crucial role of dietary cholesterol in delivering the “second hit” for NASH onset, in context of moderate dietary fat administration (45% of total calories from fat) [77]. In this study, addition of a moderate level of cholesterol in HF elicits the onset of hepatocellular damage and inflammation through activation of the inflammasomes response, while neither dietary cholesterol nor HF alone produced the NASH phenotype. Importantly, addition of cholesterol to HF resulted in blunted adaptation of mitochondrial metabolism to HF and markedly reduced mitochondrial biogenesis, effects paralleled by a decrease in PGC-1*α* and TFAM expression levels [77]. Moreover, while hepatic inflammation recovered after removal of excess dietary cholesterol, mitochondrial functions remained hampered alongside elevated NRLP3 inflammasome protein levels, indicating slow recovery dynamics from mitochondrial damage.

Excess accumulation of free cholesterol in mitochondrial membranes emerges as a hallmark of cellular transformation, potentially fueling the metabolic derangement required for cancer cell growth and resistance to apoptosis [78].

## 4. PPARs and Mitochondrial Dysfunction, from NAFLD to HCC

### 4.1. PPAR*α*

A role for PPAR*α* in NASH pathogenesis in animal models has long been established.

PPAR*α* ^−/−^ mice fed a MCD diet developed more severe NASH than WT mice, and Wy-14,643 administration completely prevented the development of NASH in WT mice, but not in PPAR*α* ^−/−^ mice [79]. The protective effect of the PPAR*α* agonist Wy-14,643 was unexpected, since the authors had foreseen a detrimental effect of the oxidative stress produced by peroxysomal *ω*-oxidation after PPAR*α* activation. However, PPAR*α* activation also resulted in increased hepatic lipid turnover through the *β*-oxidative pathway, preventing accumulation of lipoperoxides despite peroxysomal induction [79]. The beneficial effects of PPAR*α* activation by Wy-14,643 were also confirmed in a severe NASH model with established fibrosis [80].

PPAR*α* deletion in mice results in mild, age and sex-dependent, lipid accumulation in the liver [81]. Moreover, overnight fasting results in severe hypoglycemia, hypoketonemia, and increased plasma free FA levels, impaired *β*-oxidation, and ketogenesis in PPAR*α* ^−/−^ mice [19]. As a result, HFD feeding worsens NAFLD in PPAR*α* ^−/−^ mice [19, 82]. More recently, the use of a hepatocytic specific PPAR*α* ^−/−^ mice model confirmed the protective role of PPAR*α* in NAFLD induced by MCD and short-term HFD. Interestingly, PPAR*α* ^hep−/−^ mice developed steatosis and hypercholesterolemia with aging similarly to whole body PPAR*α* ^−/−^ mice but did not become obese nor hyperglycaemic [20], confirming that hepatocytic PPAR*α* deletion by itself is a primary cause of liver steatosis.

On the other hand, in leptin deficient (ob/ob) and leptin resistant (db/db) mouse models, PPAR*α* expression was found reduced, unchanged, or increased [83]. Rate of FAO also varies greatly depending on the study. While these discrepancies could be generated by different study protocols, they may be interpreted also in the light of different PPAR*α* pools that can be differentially activated in the metabolism of dietary, versus adipose tissue-derived fatty acids.

**Figure 2:**
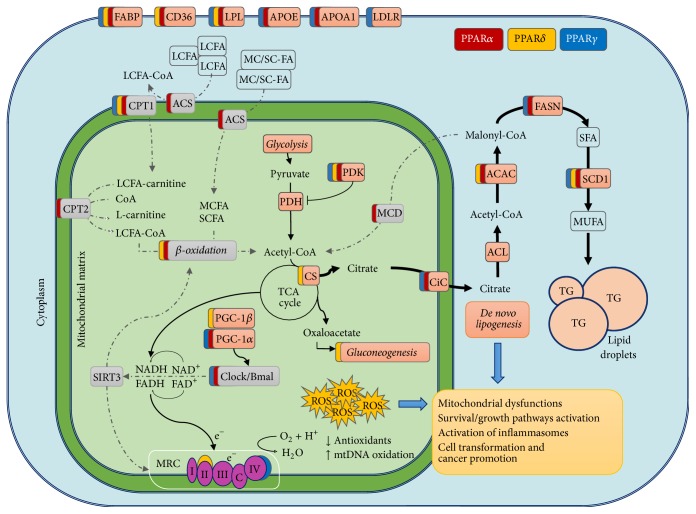
Altered mitochondrial metabolism in NASH and HCC: role of PPARs. Altered PPARs expression drives metabolic dysfunctions in the mitochondria leading to suppression of FAO, disruption of circadian rhythms, increased ROS levels, and upregulation of de novo lipogenesis. Color-coding depicts PPAR isotypes-dependency of target genes.

Since FA can bind and activate PPAR*α*, thus promoting mitochondrial and peroxysomal FAO, downregulation of PPAR*α* in NASH mice models and patients may be counterintuitive. Moreover, high FAO can increase oxidative stress; therefore stimulating PPAR*α* activity and FAO is somewhat expected to worsen the oxidative damage in hepatocytes. However, it should be recalled that although mitochondria are potentially a major source of ROS, they are also very well equipped with antioxidant defense systems. In fact, whether significant ROS production occurs in mitochondria in vivo is highly debated, and the endoplasmic reticulum is currently emerging as the major source or toxic ROS within the cell [64]. The current view is that liver triglycerides accumulation per se does not result in inflammation [84, 85]. Rather, accumulation of free fatty acids, in particular saturated fatty acids (SFA), results in marked lipotoxicity, hepatocellular damage, and inflammation [86, 87]. The onset of inflammation drives the progression from NAFLD to NASH and causes PPAR*α* downregulation by TNF*α* [88]. Moreover, TNF*α* also reduces adiponectin levels. Adiponectin promotes FAO and blunts liver gluconeogenesis signaling through AdipoR2 receptor, which promotes PPAR*α* activity [89] and depends upon PPAR*α* induction. Thus, inflammation-mediated disruption of the metabolic cross-talk between the adipose tissue and the liver may account for reduced PPAR*α* activity, mitochondrial dysfunction, and NASH development (Figure 2). A recent report by Ande and coworkers highlights the importance of the inflammatory cross-talk between adipose tissue and liver, in a sex-dependent manner, in the induction of hepatocytes mitochondrial dysfunction, NASH, and HCC development [90].

This line of view is consistent with the emerging role of PPAR*α* in the control of inflammation [12] and provides additional rationale for pharmacological induction of PPAR*α* in NASH treatment.

Reports on PPAR*α* in human NAFLD are scarce. Very recently a thorough investigation of PPARs expression in NAFLD patients was assessed by Staels' group. The expression of PPAR*α*, PPAR*β*/*δ*, and PPAR*γ* was evaluated on mRNA extracted from paired liver biopsies collected 1 year apart in 85 patients. They found a significant association between decreased PPAR*α* expression and histological severity of NASH. No correlation was found with PPAR*β*/*δ* or PPAR*γ* expression [91].

The PPAR*α* agonists peroxisome proliferators exhibit liver cancerogenic activity when chronically administered in mice. The tumor promoting activity has been related to massive proliferation of peroxisomes, with consequent oxidative stress, and to inhibition of let-7c, a microRNA that represses c-myc expression [92]. Long-term HCC development was also found to be dependent with sustained PPAR*α* activation in a transgenic model overexpressing the HCV core protein [93]. However, humans are resistant to peroxisome proliferation and indeed no association between fibrates and increased risk of any cancer has ever been found [94, 95].

Recently, PPAR*α* ^−/−^ mice were found to be more susceptible to DEN-induced HCC, and PPAR*α* anticancer activity was shown to be mediated by NF-kB inhibition [96].

Interestingly, PPAR*α* regulation of mitochondrial metabolism may be exploited for cancer treatment. Many cancer types exhibit highly glycolytic metabolism, and cancer cell's mitochondria have a strong commitment toward anabolism and cataplerosis. Since TCA intermediates are used mainly in biosynthetic reactions, mitochondria of cancer cells often have scarce OXPHOS and rely mainly on glycolysis for ATP production. Activation of PPAR*α* induces pyruvate dehydrogenase kinase 4 (PDK4) [97], which inhibits the pyruvate dehydrogenase complex, thus preventing pyruvate from glycolysis to enter mitochondria for acetyl-CoA synthesis and anaplerosis. The net result is the blockage of TCA and fatty acid synthesis, which requires acetyl-CoA, and the slowing-down of glycolytic rate [98].

Activation of PPAR*α* suppresses anaplerosis from glutamine, by repressing the expression of glutaminase and glutamate dehydrogenase, thus potentially counteracting c-myc-dependent activation of glutaminolysis in tumor [97].

Therefore, the transrepression activity of PPAR*α* on lipid biosynthesis and anaplerosis is just as relevant as its transactivation activity on FAO genes. The transrepression activity of PPAR*α* indeed impacts on mitochondria metabolism through SIRT1, by competing with ERR transcriptional pathway [99]. Interestingly, Pawlak and colleagues recently showed that the transrepression activity of PPAR*α* also regulates the inflammatory response in liver, preventing transition from NAFLD to NASH and fibrosis, and occurs independently on PPAR*α* DNA binding activity and its lipid handling properties [100].

A very recent report established a direct connection between PPAR*α*-driven FAO and hepatocyte proliferation. CyclinD1, expressed in proliferating cells and a typical protooncogene, was found to inhibit PPAR*α* expression, thereby reducing *β*-oxidation, both in normal hepatocytes and in HCC cells lines. This link was confirmed also in liver after partial hepatectomy, where induction of CyclinD1 timed with a reduction of PPAR*α* and its target genes [101].

### 4.2. PPAR*β*/*δ*

As summarized above, PPAR*β*/*δ* functions significantly overlap with PPAR*α* in peripheral tissues, while in the liver its functions are more closely related to PPAR*γ* regulated processes.

In genetic mice model of NAFLD (ob/ob), adenoviral overexpression of PPAR*β*/*δ* reduced the lipogenic program activated by SREBP-1c, via downregulation of the SREBP-1c activator insig-1, thus ameliorating hepatic steatosis [102]. Conversely, increased activation of SREBP-1c was found in PPAR*β*/*δ* ^−/−^ versus WT mice, fed either a control or ethanol liquid diet [103], suggesting that PPAR*β*/*δ* may play a role in suppressing the lipogenic pathway trough SREBP-1c.

In another study, adenoviral-mediated overexpression of PPAR*β*/*δ* in hepatocytes improved glucose utilization and hepatic insulin sensitivity. After overnight fasting, PPAR*β*/*δ* overexpressing livers had higher triglyceride and glycogen content than wild-type mice, while fatty acids and cholesterol level were similar [38]. Moreover, adenoviral-mediated overexpression in C57/BL6 mice induced SREBP-1c and PGC-1*β* expression. PPAR*β*/*δ* overexpression protected mice liver from fatty acid overload by promoting (i) FA conversion into nontoxic MUFA and (ii) FA storage into lipid droplets as triglycerides (Figure 2). As a result, activation of inflammatory pathways by FA overload was reduced in PPAR*β*/*δ* overexpressing mice fed HFD although steatosis was increased [38]. Treatment of db/db mice with the high affinity PPAR*β*/*δ* ligand GW501516 resulted in marked increase of genes involved in fatty acids synthesis and pentose phosphate pathways, promoting FA synthesis in the liver (in parallel with FA oxidation in muscle) [104].

These discrepancies are difficult to reconcile and might be related to the different mice model used, although in both genetic and dietary models PPAR*β*/*δ* has been shown to either promote or inhibit liver lipogenesis. Moreover, PPAR*β*/*δ* inhibits hepatic FGF21 expression [105], while PPAR*α* is a potent activator of FGF21 [20]. Since FGF21 is known to inhibit SREBP-1c and several other lipogenic genes in the liver [106, 107], the potential cross-talk of different PPAR isotypes on FGF21 may contribute to eliciting context-dependent effects.

Despite these striking differences, activation of PPAR*β*/*δ* consistently resulted in a beneficial effect on liver damage.

Pharmacological activation of PPAR*β*/*δ* has been explored in several rodents and human studies. Administration of PPAR*β*/*δ* agonists improved hepatic steatosis and reduced insulin resistance and hepatic inflammation [71, 108–111]. Consistently, PPAR*β*/*δ* ^−/−^ mice were prone to inflammation derived liver damage.

In humans, PPAR*β*/*δ* agonists for NASH treatment are currently under investigation in clinical trials. The first evidence in men was obtained with GW501516, which proved to be equal to the PPAR*α* agonist GW590735 in reducing plasma triglycerides levels and superior to the PPAR*α* agonist in reducing cholesterol LDL, apolipoprotein B, liver fat content, and urinary isoprostane [112]. More recently, the PPAR*β*/*δ* agonist MBX-8025 was tested in 181 dyslipidemic patients in combination with atorvastatin or alone. MBX-8025 proved effective in reducing apolipoprotein B levels, non-HDL-cholesterol, triglycerides, free fatty acids, and high-sensitive C-reactive protein [113].

PPAR*β*/*δ*-driven mitochondriogenesis has been implicated in the differentiation of hepatic-like tissue from mouse of ES cells [41]. At the early phase of differentiation, a transitory upregulation of PPAR*α* was observed, which resulted in induction of PGC-1*α* and mitochondriogenesis. Instead, the late phase of differentiation required a robust and sustained expression of PPAR*β*/*δ*, which was timely associated with albumin expression and acquisition of high mitochondrial membrane potential. PPAR*β*/*δ* agonists L165041 promoted differentiation into hepatic-like tissue that was abolished by PPAR*β*/*δ* inhibitor GSK0660 [41]. Therefore, PPAR*β*/*δ* may promote terminal hepatocyte differentiation associated with acquisition of mature mitochondria metabolism and function.

Indeed, PPAR*β*/*δ* ^−/−^ mice show a delay in liver regeneration after partial hepatectomy, associated with lack of Akt activation, lack of induction of glycolytic and lipogenic genes, and suppression of E2F transcription factors activation [114].

Interestingly, PPAR*β*/*δ* was associated with nonproliferating hepatocytes in a gene signature analysis of nuclear receptor in proliferating livers and HCC [115]. The authors analyzed the expression of all 49 members of the nuclear receptor superfamily in regenerating mouse liver and PPAR*β*/*δ* (together with TR*α* and FXR*β*) was found consistently downregulated throughout the process. PPAR*β*/*δ* was found significantly reduced in a small series of HCC with respect to the surrounding nontumoral tissue and the PPAR*β*/*δ* agonist GW501516 suppressed CyclinD1 expression and cell proliferation in Hepa1-6 cells [115]. However, whether PPAR*β*/*δ* agonists suppress HCC cells growth is still controversial [116, 117]. Both PPAR*β*/*δ* and PPAR*γ* have been implicated in mediating beta-catenin-Tcf/lef signaling [118].

Recently, PPAR*β*/*δ* was identified as a target gene of FHL2, a tumor suppressor gene also involved in hepatocellular carcinoma [119, 120].

### 4.3. PPAR*γ*

The effectiveness of the insulin sensitizers TZD in ameliorating the lipidemic profile, inflammation, and steatosis in T2DM patients is well established. Several clinical trials have explored the potential of TZDs in the treatment of NASH and have recently been reviewed [121, 122].

A recent meta-analysis of RCT on TZD and NASH (3 with pioglitazone, 1 with rosiglitazone) confirmed the effectiveness of TZD in improving steatosis, necroinflammation, and hepatocyte ballooning [123]. A significant improvement in fibrosis was obtained only when the analysis was restricted to the pioglitazone studies only. Rosiglitazone failed to improve necroinflammation, ballooning, and fibrosis in the 1-year FLIRT trial [124] and even when treatment was extended for additionally 2 years [125]. Combinatory treatment of rosiglitazone with metformin or losartan did not improve the histological endpoint versus rosiglitazone alone [126]. A very recent report suggests that rosiglitazone administration may exert opposite outcome on liver steatosis depending on liver PPAR*γ* expression levels: RGZ worsen steatosis in PPAR*γ* overexpressing mice fed a HFD and protected mice with low PPAR*γ* expression level [121, 127].

PPAR*γ* is indeed markedly overexpressed in the liver of obese patients with NAFLD and NASH, and its expression positively correlates with plasma insulin, HOMA-IR, and SREBP1-c mRNA levels and inversely correlates with adiponectin [128]. High PPAR*γ* levels, in particular of PPAR*γ*2, promotes de novo lipogenesis and liver steatosis and is associated with HFD feeding in mice [129–131]. However as recalled above, induction of PPAR*γ* by TZD, in particular pioglitazone, ameliorates steatosis and NASH. This discrepancy may be interpreted in the light of the double nature of PPAR*γ* target genes, which comprises both genes of de novo lipid synthesis and mitochondrial genes promoting FAO [132]. Moreover, pioglitazone also binds and activates PPAR*α* with low potency [133], which could explain its better performance than rosiglitazone in ameliorating steatosis. Mechanistically, induction of PPAR*γ* in steatotic hepatocytes may serve as a protective mechanism to reduce liver FFA levels by storing them as less toxic triglycerides [134, 135]. Therefore, the prosteatotic action of PPAR*γ* [136] may not be entirely detrimental. However, excess triglyceride accumulation eventually results in hepatocyte ballooning and necroinflammation, promoting transition to NASH.

The role of PPAR*γ* in hepatocellular carcinoma is still debated. A large body of literature on PPAR*γ* and cancer was produced using TZD, which eventually were proved to have several anticancer pleiotropic effects also independently of PPAR*γ* [137–140].

We and others have investigated the role of PPAR*γ* on hepatocarcinogenesis in mice harboring a hepatocyte specific deletion of PPAR*γ* gene (PPAR*γ* ^hep−/−^ mice). Yu and colleagues found increased DEN-induced HCC in mice lacking one PPAR*γ* allele, thus suggesting a tumor-suppression function for PPAR*γ* [141]. Moreover, RGZ reduced HCC development in DEN-treated WT mice but not in PPAR*γ* ^+/−^ mice [141]. Using a transgenic model of HBV-related HCC, we found that RGZ or PGZ effectively reduced HCC onset [142]. Strikingly, TZD treatment resulted more effective in PPAR*γ* ^hep−/−^ mice than in WT mice [142], highlighting that (i) TZD antitumor activity is independent of PPAR*γ*; (ii) PPAR*γ* expression reduced TZD activity; therefore in this model PPAR*γ* may support, rather than inhibiting, tumor growth.

As the master regulator of adipogenic differentiation, PPAR*γ* has been described to promote differentiation programs in a variety of tumor cell types [143, 144], inducing cell-cycle arrest [145], apoptosis/anoikis [146–148], and inhibiting EMT [149, 150], angiogenesis [151], and metastasis [152].

However, several lines of evidence also support the notion that this nuclear receptor may support the growth in several cancer types. Conflicting results have been reported in breast cancer model. Recently, Avena et al. showed that breast cancer growth was inhibited by PPAR*γ* overexpression epithelial cancer cells but promoted by PPAR*γ* overexpression in cancer associated stroma [153]. The authors identify the tumor promoting role of PPAR*γ* in the metabolic symbiosis between stoma and epithelial cancer cells, where cancer associated fibroblasts provided intermediates for mitochondrial metabolism to cancer cells [153]. Moreover, increased de novo lipogenesis, that is promoted by PPAR*γ*, is now recognized as a metabolic hallmark of cancer cell [154], including HCC [155–159] (Figure 2). Indeed, de novo lipogenesis is activated downstream of the Akt/mTOR pathway, one of the most common signaling pathways altered in cancer. Forced activation of Akt/mTOR induces liver cancer [160, 161], a process mediated at least in part by activation of FASN [155, 156]. Consistently, inactivation of FASN was recently shown to completely inhibit Akt-driven HCC in mice [158]. Importantly, FASN is not oncogenic per se. However, when the PI3K/Akt/mTOR pathway becomes hyperactive, the induction of the de novo lipogenesis is a requisite for supporting cancer cell growth. Importantly, PPAR*γ* is a direct transcriptional target of mTORC1 [162]. Moreover, in PTEN null mice PPAR*γ* was found to directly induce the expression of key glycolytic gene HK and oncogenic PKM2, inducing hepatocyte steatosis, hypertrophy, and hyperplasia [163].

Therefore, PPAR*γ* may inhibit or promote HCC development depending on the metabolic context, the cell type expressing it, the oncogenic signaling pathways involved, and dietary or pharmacological treatment. It is however conceptually very attractive to explore the therapeutic potential interference with the cancer cell lipid handling capacity, through modulation of mitochondrial FA, ketogenesis, and lipogenesis, as an integrated anticancer approach.

## 5. PPARs and Circadian Regulation of Mitochondria Metabolism

Many processes of our metabolism and physiology are regulated by circadian clocks, endogenous time-tracking systems that coordinate daily rhythms of rest, activity, feeding behavior, energy utilization, and storage. Although circadian rhythms are endogenous they respond to external stimuli, which include light, temperature, and redox cycles [164]. Circadian regulation is coordinated by the suprachiasmatic nucleus in the brain, but most peripheral organs contain their own independent pacemakers [165]. At a cellular level these oscillations are driven by transcriptional feedback loops associated with changes in chromatin remodeling, mRNA processing, protein turnover, and activity [166–169]. Main factors that control circadian rhythmicity in the cells include BMAL1 and CLOCK (“activators”) and CRYs and PERs (“inhibitors”). Their effects are tissue-specific and in the liver they control approximately 10% of the transcriptome [170], influencing metabolic pathways by modifying the expression or activity of key enzymes and transporters involved in lipid, glucose, and mitochondrial oxidative metabolism. Reciprocally, intracellular metabolites and transcriptional factors modulate CLOCK activity in response to the energy status.

Circadian dysregulation of lipid metabolism, ROS production, and cell-cycle control is linked to various pathological conditions including metabolic syndrome, diabetes, chronic liver diseases, and cancer [171–173].

### 5.1. Clock and Lipid Metabolism: Regulation of PPARs and Mitochondrial Functions

The redox state of the cell also seems to play an important part in the rhythmicity of metabolism, especially in the mitochondria. NAD+ levels oscillate and are under direct control of clock transcription factors that upregulate the rate-limiting enzyme in NAD^+^ biosynthesis, NAMPT (nicotinamide phosphoribosyl transferase). In mitochondria NAD+ activates SIRT3, an important regulator of intrinsic mitochondrial function including FAO. In the cytoplasm NAD+ activates SIRT1 that operates a small feedback regulating Clock and Bmal. Disruption of circadian rhythms in mice leads to defects in mtFAO and decreased OCR mainly through deregulation of NAD+ dependent SIRT3 activity [174, 175] (Figure 2).

Several genes involved in lipid metabolism (such as SREBP, HMGCoAR, and FAS) are modulated by PPAR*α* and display circadian fluctuations that are lost in PPAR*α*-KO mice [176, 177].

PPAR*α* is a direct transcriptional target of BMAL1 and CLOCK [178–180] and in the rodent liver operates a feedback loop binding BMAL1 and REV-ERB*α* gene promoters. BMAL1-KO and CLOCK-mutant mice display abolished PPAR*α* oscillation and decreased expression in the liver, whereas PPAR*α*-KO mice display altered oscillation of PER3 and BMAL1 [181]. Moreover, administration of PPAR*α* agonists fenofibrates upregulates the expression of* Bmal1* in mouse liver [180].

Fatty acids are known to be PPAR*α* activators, binding directly to the transcriptional factor. Interestingly, hepatic fatty acids are also produced in a circadian manner by acyl-CoA thioesterases (ACOTs) and lipoprotein lipases (LPLs).

The expression of both enzyme families displays circadian rhythmicity; it is regulated by PPAR*α* and can in fact be induced by WY14643. Moreover, silencing members of ACOTs lead to a downregulation of Cyp4a10 and Cyp4a14, PPARa targets [182–186].

Another clock controlled gene, Nocturnin, binds to PPAR*γ* modulating its transcriptional activity [187], and PPAR*γ* systemic inactivation in mice leads to impaired rhythmicity of the canonical clock genes in liver and adipose tissues [188]. PGC-1*α* is also rhythmically expressed in mouse liver and muscle, upregulates circadian factors BMAL1, CLOCK, and REV-ERB*α* [189], and modulates the length of circadian oscillations by controlling Bmal1 transcription in a REV-ERB-dependent manner. Mice lacking PGC-1*α* show abnormal circadian rhythms and altered expression of metabolic genes [189]. Interestingly, circadian regulation was lost also in mice lacking PGC-1*β*, but this resulted in markedly decreased activity during the dark cycle, as opposed to the hyperactive PGC-1*α* KO mice [190] (Figure 2).

The liver-specific deletion of PPAR*δ* in mice showed that it is involved in the temporal regulation of several lipogenic genes, such as fatty acid synthase (FAS) and acetyl-CoA carboxylase 1 and acetyl-CoA carboxylase 2 [40]. BMAL1 also induces the expression of REV-ERB*α*, a nuclear receptor that downregulates BMAL1 itself, operating a negative feedback, and upregulates the expression of a liver-specific microRNA: miR-122 [191]. miR-122 is also involved in lipid metabolism in mouse liver [192] and PPAR*δ* was proven to be one of its targets, suggesting that PPAR*δ* plays a role in hepatic circadian regulation [193].

The circadian regulation of mitochondrial metabolism is still in its early days. Using a MS-based proteomic approach, the expression of rate-limiting enzymes and metabolites in mitochondria was quantitatively evaluated throughout the day [194]. Many key mitochondrial enzymes involved in carbohydrates and lipid metabolism were found to peak in the early morning period and to be regulated by PER2/3 proteins. Mitochondrial respiration displayed an oscillatory behavior, peaking several times of the day. In mice KO for Per2/3, as well as in those fed a HFD, period protein oscillation was lost, together with OXPHOS oscillation [194].

### 5.2. Circadian Disturbances in Liver Disease

It is now clear that circadian rhythms are fundamental in liver physiology and their disruption is observed in many hepatic pathologic conditions, such as NASH, NAFLD, ALD, and HCC [110, 172, 195–198].

In a mouse model of NASH it was found that HFD induces the susceptibility to develop NASH through desynchronized Clock gene expression and altered cellular redox status, accompanied by reduced sirtuin abundance [197]. HFD in mice is sufficient to induce the loss of circadian fluctuations of insulin secretion [199]. Conversely, BMAL1 whole body-KO mice and Clock-mutant mice display hepatic steatosis, obesity, hypoinsulinemia, and increased glucose intolerance [200].

The molecular alterations found in the liver of HFD-fed mice include loss of oscillation or phase advance of rhythmicity of many genes involved in lipid and mitochondrial metabolism (such as NAMPT, acetyl-coenzyme A synthetase, and ornithine decarboxylase 1) and gain of oscillation of other genes such as PPAR*γ* and its targets [201]. This transcriptional reprogramming relies on changes in the oscillation and chromatin recruitment of PPAR*γ* that also induces the oscillation of* Cidec* (cell death activator CIDE-3) [201], a protein that is substantially elevated in the livers of the obese ob/ob mice [202]. Administration of GW9662, a specific PPAR*γ* antagonist, into HFD-fed animals produced a decrease in PPAR*γ*-induced Cidec expression [201]. The expression of another known PPAR*γ* target, pyruvate carboxylase (Pcx), an important regulator of hepatic gluconeogenesis, was significantly elevated and rhythmic in livers of HFD-fed mice [201]. In Nocturnin-KO mice fed with HFD, liver PPAR*γ* oscillation was abolished, accompanied by a reduced expression of many genes related to lipid metabolism and resistance to hepatic steatosis [203].

Accumulating evidence supports the importance of the disruption of circadian rhythms in various types in cancer. Specifically, in HCC patients, low expression of clock genes was observed in the cancerous tissue, but not in the noncancerous liver tissue, and correlated with tumor size and tumor grade [204]. A number of mechanisms may explain the circadian control on HCC. For example, it was found that DEN exposure in mice is associated with circadian disturbance, suggesting that liver clocks are involved in the carcinogenesis [196]. Mutations and polymorphisms of the clock proteins are being screened to assess their association with HCC. Interestingly, a functional polymorphism of PER3 was recently associated with a lower risk of death in HCC patients treated with TACE [205].

## 6. Perspectives and Conclusions

It is now clear that expression or activation of nuclear receptors, including PPARs, is not sufficient to predict their biological output. The net effect of a nuclear receptor activation in a given cell actually depends on the context of coactivators, corepressors, dimerization events, availability of endogenous/synthetic ligands, posttranslational modifications, competition, and interactions with other NRs. This led to the development of partial agonist selective PPAR modulators (SPPARMs), a second generation of PPAR agonists able to selectively activate a subset of target genes downstream a specific PPAR isotype.

K-877 is a SPPAR*α*M currently being tested in dyslipidemic patients that exhibits higher lipid lowering activity than fibrates and has a favorable risk profile [206, 207]. INT-131, SPPAR*γ*M, has potent glucose lowering effects not associated with TZD side-effects [208].

A different approach to PPAR modulation is to simultaneously activate, with different potency, more than one isotype: dual PPAR agonist or pan-agonists are currently under investigation. The dual PPAR*α*/*δ* agonist GFT-505 is proving effective in reducing plasma triglyceride levels, improving insulin sensitivity, and increasing HDL-cholesterol in obese patients [209, 210] and showed promising results in mice model of NASH [211]. Very recently a phase 2 multicenter randomized controlled trial, enrolling 274 subjects with histologically proven NASH, showed that GFT505 produces a dose-dependent improvement in histology of patients with NASH [212].

As we gain knowledge of the metabolic circadian regulation and of its disruption in disease, an entire new area of intervention begins to emerge. Modulation of amplitude and phase of PPARs circadian regulation could be exploited to drive complex metabolic remodeling of mitochondrial metabolism in NASH and cancer models. Finally, the integration of the above-mentioned approaches with the metabolic and genetic profiling of cancers holds the promise for new therapeutic approaches that can selectively target the fuel requirements of HCC.

## Abbreviations

ACAA2: Acetyl-CoA acyltransferase 2
ACADM: Medium-chain specific acyl-CoA dehydrogenase
ACADs: Acyl-CoA dehydrogenases
ACADVL: Very long-chain specific acyl-CoA dehydrogenase
ACC1 and ACC2: Acetyl-CoA carboxylase 1 and acetyl-CoA carboxylase 2
ACS: Acetyl-coenzyme A synthetase
AdipoR2: Adiponectin receptor 2
MAL1: Aryl hydrocarbon receptor nuclear translocator-like protein 1
CPT-1 and CPT-2: Carnitine palmitoyl transferase 1 and carnitine palmitoyl transferase 2
DAMPs: Damage-associated molecular pattern
ERRalpha: Estrogen related receptor alpha
ETC: Electron transport chain
FAO: Fatty acid oxidation
FAS: Fatty acid synthase
FAT/CD36: Fatty acid translocase
FHL2: Four and a half LIM domains protein 2
FXR: Farnesoid X receptor
GK: Glycerol kinase
GLUT2: Glucose transporter 2
GLUT-4: Glut transporter 4
HADHA and HADHB: Trifunctional enzyme subunit alpha and beta
H-FABP: Heart-type fatty acid-binding protein
HMGCoAR: 3-hydroxy-3-methylglutaryl-CoA reductase
HNF-4: Hepatocyte nuclear factor 4-alpha
IRS-1, IRS-2: Insulin receptor substrate 1 and insulin receptor substrate 2
JNK: Jun N-terminal kinase
LC-FA: Long-chain fatty acids
LPL: Lipoprotein lipase
LXR: Liver X receptor
M/S-FA: Medium/short chain fatty acids
MAPK: Mitogen-activated protein kinase 1
MCD: Methionine and choline deficient diet
MDA: Malondialdehyde
MTP: Mitochondrial trifunctional protein
MUFA: Monounsaturated fatty acids
NAD+: Nicotinamide adenine dinucleotide
NAMPT: Nicotinamide phosphoribosyltransferase
NCOR: Nuclear receptor corepressor 1
NF-*κ*B: Nuclear factor NF-kappa-B
PAMPs: Pathogen associated molecular patterns
PDK4: Pyruvate dehydrogenase kinase 4
PEPCK: Phosphoenolpyruvate carboxykinase
PGC-1*α*/*β*: Peroxisome proliferator activated receptor gamma coactivator 1 *α*/*β*
PI3K: Phosphatidylinositol 3-kinase
PKM2: Pyruvate kinase M2
PTEN: Phosphatidylinositol 3,4,5-trisphosphate 3-phosphatase and dual-specificity protein phosphatase
REV-ERBa: Nuclear receptor subfamily 1 group D member 1
SCD1: Acyl-CoA desaturase 1
SFA: Saturated fatty acids
SIRT-1 andSIRT-3: NAD-dependent protein deacetylase sirtuin-1 andNAD-dependent protein deacetylase sirtuin-3
SOD: Superoxide dismutase
TR*α*: Thyroid hormone receptor alpha (TR-alpha)
UCP-1, UCP-2, and UCP-3: Mitochondrial uncoupling protein 3
4-HNE: 4-hydroxynonenal.
